# Evaluation of a Neonatal Resuscitation Training Programme for Healthcare Professionals in Zanzibar, Tanzania: A Pre-post Intervention Study

**DOI:** 10.3389/fped.2021.693583

**Published:** 2021-06-28

**Authors:** Xiang Ding, Li Wang, Mwinyi I. Msellem, Yaojia Hu, Jun Qiu, Shiying Liu, Mi Zhang, Lihui Zhu, Jos M. Latour

**Affiliations:** ^1^International Affairs, Hunan Children's Hospital, Changsha, China; ^2^Training and Research, Mnazi Mmoja Hospital, Zanzibar, Tanzania; ^3^Nursing School, Hunan University of Chinese Medicine, Changsha, China; ^4^Editing Office, Journal of Clinical Pediatric Surgery, Hunan Children's Hospital, Changsha, China; ^5^Neonatal Department, Hunan Children's Hospital, Changsha, China; ^6^Nursing Department, Hunan Children's Hospital, Changsha, China; ^7^Faculty of Health, University of Plymouth, Plymouth, United Kingdom

**Keywords:** newborn, neonatal resuscitation, training, knowledge, skills, healthcare professionals, mortality

## Abstract

**Background:** Neonatal mortality rates remain high in Sub-Saharan African countries. Improving the newborn resuscitation skills of healthcare professionals is important in addressing this challenge. The aim of this study was to evaluate a neonatal resuscitation training programme delivered over a two-year period for healthcare professionals in Zanzibar, Tanzania.

**Methods:** A pre- and post-intervention study was designed. We delivered neonatal resuscitation training over a 2-day period in 2017 and 2 days of refresher training in 2018. Knowledge was evaluated by a self-designed survey (11 items with a total score of 22) before and after the two training periods, and skills were evaluated by a skills checklist (six domains with 25 items with a total score of 50) completed by the trainers based on their observations. Statistical analysis included differences in the knowledge and skills scores before and after the training sessions and between the two periods.

**Results:** A total of 23 healthcare professionals participated and completed both neonatal resuscitation training sessions. The knowledge mean scores before and after the training in 2017 increased from 9.60 to 13.60 (95% CI: −5.900; −2.099, *p* < 0.001), and in 2018, the scores increased from 10.80 to 15.44 (95% CI: −6.062; −3.217, *p* < 0.001). The mean knowledge scores post-training over time were 13.60 in 2017 and 15.44 in 2018 (95% CI: −3.489; 0.190, *p* = 0.030). The resuscitation skills performance between the two time periods increased from a mean of 32.26 (SD = 2.35) to a mean of 42.43 (SD = 1.73) (95% CI: −11.402; −8.945, *p* < 0.001).

**Conclusion:** The neonatal resuscitation training programme increased the theoretical knowledge and resuscitation skills before and after the two training sessions and over time after a 9-month period. Continuous neonatal resuscitation training based on the local needs in resource-limited countries is essential to provide confidence in healthcare professionals to initiate resuscitation and to improve newborn outcomes.

## Introduction

While child mortality has improved globally over the past decades, neonatal mortality rates have remained stagnant in certain areas. The annual estimated range of child mortality decreased from 4.7 to 2.8 million, with a 40% drop between 1990 and 2013 (1, 2). Almost half of neonatal deaths occur in low- and middle-income countries. This is most prevalent in Sub-Saharan countries in Africa, including Tanzania (3). Tanzania has one of the lowest physician-to-population ratios in the world, and neonatal mortality remains high at 21 deaths per 1,000 live births (4). Although significant efforts have been made to fulfill the Sustainable Development Goals, less progress has been shown in the reduction of neonatal mortality rates to at least 12 per 1,000 live births (5, 6). High neonatal mortality is partially influenced by a lack of qualified healthcare professionals with skills in neonatal resuscitation, inadequate training, and insufficient medical resources (7). Therefore, neonatal resuscitation training programmes are a priority to reduce preventable deaths and thereby reduce neonatal mortality (8).

Birth asphyxia and failure to initiate or sustain resuscitation at birth remain the main causes of neonatal deaths worldwide (9). These data are even higher in rural locations, such as in Zanzibar hospitals, where up to 60% of neonatal deaths occur (10, 11). A survey study in five rural districts of southern Tanzania identified 219 neonatal deaths (12). The most common causes of neonatal death were prematurity (*n* = 72; 33%), birth asphyxia (*n* = 49; 22%), infection (*n* = 21; 10%), congenital abnormalities (*n* = 10; 5%), and 19 other causes (9%) (12).

Several studies have demonstrated that interventions related to improving neonatal resuscitation can increase infant survival (13–15). Furthermore, home delivery is considered a common practice in Zanzibar, and only a few skilled birth attendants are available for home delivery (16). Skilled birth attendants in Zanzibar include midwives and healthcare workers who are trained in normal delivery, pregnancy management, and referral in case of complications. Additionally, a study performed in regions of Africa, Asia and Latin America/Caribbean indicated that a short period of in-service training was significantly associated with decreased neonatal mortality (17).

The retention of knowledge and skills over time is essential in healthcare professional training and has been reported to be a challenge in limited-resource countries (18–20). A systematic review demonstrated that in four included studies, there was no or only a limited drop in knowledge and skills after a refresher newborn resuscitation training over a period between nine months and two years (21). Currently, there is limited evidence of the impact of sustained knowledge and skills as well as the frequency of repeated training and knowledge in resource-limited settings. To address this gap, we explored a strategy to improve the training of healthcare professionals in neonatal resuscitation.

In 2013, with the proposal of the ‘Belt and Road Initiative', China made a commitment to provide training opportunities to 100,000 professionals in Asian and African countries (22). Initial discussions with officials in Zanzibar revealed a need for neonatal resuscitation training (NRT) for medical and nursing staff because training resources were limited. Colleagues from the USA have already started projects to improve neonatal resuscitation knowledge (11). To meet the needs of the local staff and contribute to the improvement of medical training in Zanzibar, we developed and implemented a NRT programme for healthcare professionals in Zanzibar, Tanzania.

The aim of this study was to evaluate a NRT programme for healthcare professionals in Zanzibar. Specifically, the objective was to evaluate the acquisition and retention of healthcare professionals' neonatal resuscitation knowledge and skills.

## Materials and Methods

The study used a pre-post intervention design. The NRT was implemented in Zanzibar, Tanzania, over a 2-day period in September 2017, and nine months later, 2 days of refresher training was repeated in June 2018. Data were collected before and after the training sessions in 2017 and 2018, respectively.

### Setting

The study was conducted in the 510-bed Mnazi-Mmoja Hospital on the island of Zanzibar in Tanzania. This tertiary teaching hospital serves a population of nearly 1.4 million. This publicly funded hospital includes a pediatric department with a neonatal department with a 30-bed capacity.

### Participants and Recruitment

Study participants were selected by the Zanzibar Ministry of Health. Participants were eligible if they were healthcare staff who voluntarily wanted to participate in the NRT and were able to follow the full training days. In total, 23 professionals from three health sectors participated in both NRT programmes delivered in 2017 and 2018: 14 participants from Mnazi-Mmoja Hospital (three neonatal physicians, five neonatal nurses, six mid-wives), five participants from Makunduchi Primary Healthcare Center (two nurses and three physicians), and four participants from Kivunge Primary Healthcare Center (two physicians and two administrators).

### Neonatal Resuscitation Training

The NRT consisted of theory and hands-on simulation sessions. The training was designed and provided by five neonatal experts with English proficiency from Hunan Children's Hospital in China after exploring the participants' needs and the standard neonatal resuscitation protocols in Zanzibar. The training was based on the American Heart Association guidelines (23, 24). The curriculum of the 2-day training used in 2017 and 2018 is presented in Table 1. The hands-on simulation sessions used a trainer-trainee ratio of 1:5. The same curriculum of the NRT was provided to the same 23 participants in 2017 and 2018. All materials were provided in English, including course materials, handouts, and checklists. Two Kiswahili interpreters supported the 2-day training sessions to assure effective communication. The Chinese trainers were five neonatal physicians who were trained and authorized by the Chinese National Neonatal Resuscitation Program.

**Table 1:** Curriculum of neonatal resuscitation training.

**Date**	**Time**	**Title**	**Content/description**
Day 1	08:00–09:00	Overview and principles of resuscitation (theory classroom)	Describe the 2015 American Heart Association neonatal resuscitation guidelines of and preparation before resuscitation
	09:00–10:00	Initial steps of resuscitation (theory classroom)	The initial steps of resuscitation are to provide warmth, give the right position, clearing the airway, drying the baby, and stimulating breathing.
	10:30–12:30	Endo-tracheal intubation and laryngeal mask (theory classroom)	The indications, devices, procedures and contradictions of intubation and laryngeal mask
	14:00–17:30	Neonatal resuscitation (hands-on simulation)	Divided into 4 groups for ABCD, each group had 2 teachers and 5–6 students, giving the scenario training followed by initial step to intubation
Day 2	08:00–09:00	Chest compression (hands-on simulation)	The indications, devices, procedures and contradictions of chest compression
	09:00–10:00	Use of resuscitation devices (hands-on simulation)	The indication for starting mechanical ventilation, initial breaths, assisted ventilation and end-expiratory pressure
	10:30–11:30	Medications and consideration (theory classroom)	The indications, types and contra-indications of medication; brief explanation for special conditions (meconium aspiration, congenital hernia diaphragm, withholding resuscitation, etc.)
	11:30–12:30	Resuscitation of babies born preterm (theory class room)	The special items for preterm birth (temperature control, oxygen concentration, etc.)
	14:00–15:30	Neonatal resuscitation (hands-on simulation)	Divided into 4 groups for ABCD, each group had 2 teachers and 5–6 students, giving the scenario training for all the contents
	16:00–18:00	Neonatal resuscitation evaluation (classroom)	23 participants were divided into 8 groups for evaluation

### Data Collection

Prior to training, each participant completed a demographic questionnaire in 2017 collecting the participants' characteristics: gender, working experience in years, and profession. The knowledge questionnaire and the skills assessment form were designed and validated by 10 neonatal physicians certified by the Chinese National Neonatal Resuscitation Program linked to the Resuscitation Guidelines of the American Heart Association. The questionnaire and assessment form have been used previously in other medical settings in limited-resource countries. These forms were discussed and agreed upon by our colleagues in Zanzibar.

Knowledge was tested by a self-designed questionnaire (Supplementary Material 1). The test included 11 multiple choice questions (only one answer was correct out of the four answer options) related to general knowledge of neonatal resuscitation. For any correct answer, a score of 2 points was given. The total score range of the questionnaire was 0–22.

The skill performance assessment form consisted of six domains with a total of 25 observation items assessing resuscitation procedures (Supplementary Material 2). Every correctly performed item received two points. The total score of the skills assessment was 50 points. Trainers assessed the participant's behavior and completed the skills performance assessment form.

Data were collected by the five trainers who were certified neonatal resuscitation trainers. The five trainers were the same physicians in 2017 and 2018. Each participant completed the knowledge test pre- and post-training in both years. The skills were assessed in a session after the hand-on simulation training.

### Data Analysis

Data were analyzed using IBM SPSS version 25.0. Participants' characteristics were descriptively analyzed using frequencies. The item scores of the knowledge questionnaire were calculated and presented as the mean and standard deviation (SD). When comparing the pre- and post-knowledge item scores, the frequency (%) of the total participants with correct answers was calculated, and an independent sample *t*-test was used. The chi square test and Fisher's exact test were used to evaluate the differences in knowledge in the 2-year period. Finally, the independent sample *t*-test was used to analyse the domains of neonatal resuscitation skill performance in 2017 and 2018.

### Ethics

This study was approved by the Medical Ethics Committee of Hunan Children's Hospital (HCHLL-2018-03). The training programme was supported by the Zanzibar Ministry of Health. The study participants received the study procedures, including neonatal resuscitation training details and questionnaires, before the training. Informed consent was obtained by the participants via verbal informed consent, as written consent was waived by the Medical Ethics Committee of Medical Ethics Committee of Hunan Children's Hospital. All methods were performed in accordance with the relevant guidelines and regulations, including the Declaration of Helsinki.

## Results

In total, 23 participants completed both training programmes in 2017 and 2018. Of these, 18 were female, and their professions were physicians (*n* = 8), nurses (*n* = 13), and hospital administrators (*n* = 2). Work experiences were 1 year (*n* = 10), 2 years (*n* = 3), 3 years (*n* = 7), and 4 years (*n* = 3).

Knowledge was tested before and after both training sessions in 2017 and 2018. In 2017, the mean knowledge scores increased from 9.60 to 13.60 (mean difference −4.00, 95% CI: −5.900; −2.099, *p* < 0.001), and in 2018, the scores increased from 10.80 to 15.44 (mean difference −4.64, 95% CI: −6.062; −3.217, *p* < 0.001). Over time, the mean difference in the knowledge scores post-training in 2017 was 13.60 and in 2018 was 15.44 (mean difference −1.84, 95% CI: −3.489; −0.190, *p* = 0.030) (Table 2).

**Table 2:** Knowledge scores before and after training in 2017 and 2018.

	**n**	**2017** **mean (SD)**	**2018** **mean (SD)**	**Mean difference** **(95% CI)**	***p*-Value**
Pre-training	23	9.60 (2.89)	10.80 (3.05)	−1.20(−3.179; 0.779)	0.223
Post-training	23	13.60 (3.74)	15.44 (1.78)	−1.84(−3.489; −0.190)	0.030
Mean difference(95% CI)		−4.00(−5.900; −2.099)	−4.64 (−6.062; −3.217)		
*p*-Value		<0.001	<0.001		

The correct answer ratios and the improvement ratios of the knowledge tests pre- and post-training at the two time points were analyzed. All knowledge items improved from pre- to post-training (Table 3). Over time, the knowledge of four items was sustained at 100%: neonatal resuscitation assessment signals, evaluation of the effectiveness of positive pressure, indications of effective neonatal resuscitation, and appropriate compression depth (Table 4). The correct response to the question on “Consideration of long-term (>2 min) face-mask safety during non-invasive positive pressure ventilation” increased from 13% to 39% in 2017 and from 17% to 100% in 2018 (Table 4). This indicated an improvement of 61% over time (*p* < 0.001).

**Table 3:** Correct answers of knowledge tests before and after training in 2017 and 2018 (*n* = 23).

	**2017**				*****χ***^**2**^**	***P-*value**	**2018**				*****χ***^**2**^**	***P-*value**
	**Pre-training**		**Post-training**				**Pre-training**		**Post-training**			
	***n***	**%**	***n***	**%**			***n***	**%**	***n***	**%**		
Airway management	13	57	23	100	12.78	<0.001	18	78	23	100	5.61	0.018
Neonatal resuscitation assessment signals	20	87	23	100	3.21	0.073	19	83	23	96	4.38	0.036
Evaluate the effectiveness of positive pressure	8	35	16	70	5.58	0.018	19	83	23	100	4.38	0.036
Neonatal oxygen concentration	10	43	19	83	7.56	0.006	7	30	21	91	17.89	<0.001
Respiratory rate under positive pressure ventilation	8	35	15	65	4.26	0.039	9	39	23	100	20.13	<0.001
Consideration of long-term (>2 min) face-mask safety during non-invasive positive pressure ventilation'	3	13	9	39	4.06	0.044	4	17	23	100	32.37	<0.001
Indications of effective neonatal resuscitation	18	78	23	100	5.61	0.018	16	70	23	100	8.26	0.004
Neonatal resuscitation compression and ventilation ratio	11	48	19	83	6.13	0.013	5	22	23	100	29.57	<0.001
Appropriate compression depth	19	83	23	100	4.38	0.036	13	57	23	100	12.78	<0.001
Appropriate routes of epinephrine administration	9	39	15	65	3.13	0.077	14	61	22	96	8.18	0.004
Neonatal fluid resuscitation	6	26	14	61	5.66	0.017	7	30	22	96	20.99	<0.001

*n, participants with correct answers; %, correctness rate*.

**Table 4:** Knowledge improvement rates before and after training in 2017 and 2018 (n = 23).

	**Theoretical knowledge**					*****χ***^**2**^**	***P-*value**
	**2017**		**2018**		**Improvement rate (%)**		
	***n***	**%**	***n***	**%**			
Airway management	23	100	23	100	0	/	/
Neonatal resuscitation assessment signals	23	100	23	100	0	/	/
Evaluate the effectiveness of positive pressure	16	70	23	100	30	6.18	0.013
Neonatal oxygen concentration	19	83	21	96	10	0.76	0.381
Respiratory rate under positive pressure ventilation	15	65	23	100	35	7.61	0.006
Consideration of long-term (>2 min) face-mask safety during non-invasive positive pressure ventilation	9	39	23	100	61	20.13	<0.001
Indications of effective neonatal resuscitation	23	100	23	100	0	/	/
Neonatal resuscitation compression and ventilation ratio	19	83	23	100	17	4.38	0.036
Appropriate compression depth	23	100	23	100	0	/	/
Appropriate routes of epinephrine administration	15	65	22	96	32	6.77	0.009
Neonatal fluid resuscitation/expansion	14	61	22	96	36	8.18	0.004

n, participants with correct answers; %, correctness rate; improvement rate = [(pre-training scores- post-training scores)/full scores of each item]*100.

Table 5 presents the scores of the participants' skill performance after the theoretical and hands-on training. Overall, the total mean scores of the six domains increased from 32.26 (SD = 2.35) in 2017 to 42.43 (SD = 1.73) in 2018 (Table 5). Improvement ratios increased most in the domains: initial neonatal resuscitation (36%), endotracheal intubation and chest compression (33%), and drug administration (33%).

**Table 5:** Comparison of skill performance in 2017 and 2018 (n = 23).

	**Maximum score**	**2017** **Mean (SD)**	**2018** **Mean (SD)**	**Improvement** **rates (%)**	**Mean difference** **(95% CI)**	***p*-value**
Resuscitation preparedness and rapid assessment	4	2.78 (0.42)	3.33 (0.41)	20	−0.543 (−0.792, −0.294)	<0.001
Initial neonatal resuscitation (warming, positioning, airway clearance, oxygenation, etc.)	10	6.34 (0.93)	8.65 (0.73)	36	−2.304 (−2.802, −1.806)	<0.001
Positive pressure ventilation (indications assessment, air-mask ventilation procedures, etc.)	12	7.65 (0.83)	10.00 (0.84)	31	−2.347 (−2.844, −1.851)	<0.001
Endo-tracheal intubation and chest compression	12	7.39 (0.78)	9.84 (0.97)	33	−2.456 (−2.980, −1.932)	<0.001
Drug administration	10	6.48 (0.85)	8.60 (0.88)	33	−2.130 (−2.642, −1.617)	<0.001
Final evaluation	2	1.61 (0.50)	2.00 (0.00)	24	−0.391 (−0.601, −0.181)	0.0005
Total scores	50	32.26 (2.35)	42.43 (1.73)	32	−10.173 (−11.402, −8.945)	<0.001

Table 6 presents the 10 key items of inadequate neonatal resuscitation skills in 2017 and 2018. In 2017, 74% of the participants showed poor performance on aspects of “inadequate preparedness” and “unfamiliar with epinephrine administration”, 70% of the participants failed in “endotracheal intubation”, and 65% of the participants failed in the “inappropriate body position” and “location of chest compressions”. However, “endotracheal intubation” and “inappropriate body position” remained common items affecting resuscitation skills in 2018, but these declined to 39% and 35%, respectively.

**Table 6:** Key items of inadequate skill performance.

**Skill performance items**	**2017**		**2018**	
	***N***	**%**	***n***	**%**
Inadequate preparedness	17	74	6	26
Inappropriate body position	15	65	8	35
Evaluation of oxygen delivery	18	61	3	13
Artificial Positive Pressure maneuver	13	57	7	30
Endo-tracheal intubation	16	70	9	39
Location of chest compressions	15	65	5	22
Insufficient compression frequency	13	57	7	30
Unfamiliar with epinephrine administration	17	74	4	17
Improper use of volume expanders	14	61	5	22
Final evaluation	10	43	0	0

*Percentages represent the number of participants (total n = 23) who scored 1 (incorrect, incomplete, or wrong) on the skills performance form*.

## Discussion

We evaluated the implementation of a NRT programme in the resource-limited setting of Zanzibar and assessed the acquisition and sustainability of neonatal resuscitation competencies among healthcare professionals. Theoretical knowledge and skills performance improved in most categories, which supports the effect of neonatal training over a two-year period. The post-training scores of theoretical knowledge and skill performance in 2018 were higher than those in 2017. This indicates that refresher courses might improve knowledge compared to one course only. Studies from other resource-limited countries, such as Bangladesh and Ethiopia, reported that improvements in knowledge and skills were observed at 1 year and 18 months after newborn health training, respectively (25, 26). Our results are consistent with those of Bang et al. and Drake et al. who documented that ongoing training programmes are more effective and necessitated, as these can improve knowledge and skill retention, especially in resource-limited countries (22, 27). A pre- and post-intervention study testing a long-term newborn training programme in Somalia over a two-and-a-half-year period (including an 18-month follow-up) demonstrated that some skills did not improve (28). Issues such as hand hygiene and discharge education did not improve over time, and the authors suggested that different training approaches were needed to address the local needs. Developing and implementing training in resource-limited settings remains a challenge and should be coordinated collaboratively with colleagues outside these settings.

The interval between the two NRT sessions in our study was nine months. Despite the paucity of evidence for an optimal resuscitation training interval, effective strategies for teaching, assessing, and maintaining knowledge and skills are recommended (29, 30). A recent systematic review looking at the effectiveness of neonatal simulation sessions demonstrated that the long-term retention of skills is related to training time and duration (31). However, the effects of high-fidelity simulation training of healthcare professional skills in real life situations has not yet been demonstrated by rigorous randomized controlled trials (31, 32).

Our study indicated that several competencies in neonatal resuscitation are difficult to maintain over time by healthcare professionals. Hence, we revised and adjusted the training content based on the local practical context and the needs of the local healthcare professionals. The results indicate that ongoing support is needed for local healthcare professionals' skills and work collaboratively together to reach the Sustainable Development Goals in 2030 (5). The main causes of neonatal deaths are prematurity, asphyxia, and sepsis (33, 34). A pre- and post-experimental study of the NRT programme may not directly lead to a decrease in neonatal death. However, full competence and proficiency in neonatal resuscitation might contribute to decreased neonatal mortality and neonatal asphyxia (32, 35).

Neonatal care and resuscitation in Zanzibar have received increasing attention from healthcare professionals from other countries, such as the USA, in delivering the Helping Babies Breathe (HBB) programme to midwives (36). The HBB programme has been implemented with a limited budget and the knowledge and skills of midwives retained over a 6-month post-training time. Our initiative contributed to the knowledge and skills of several different healthcare professionals from different healthcare settings in Zanzibar. It is indeed important to establish a long-term relationship among international colleagues and experts in neonatal resuscitation to work collaboratively toward the target of the Every Newborn Action Plan of the Sustainable Development Goals trying to reduce neonatal mortality to 10 or less per 1,000 live births by 2035 (37). Therefore, successful neonatal resuscitation training programme need to focus on the sustainability of competence and translate the generated knowledge into clinical practice to tackle medical problems and reduce neonatal mortality and morbidity (38). An example is the recently tested Rapid Feedback for quality Improvement in Neonatal rEsuscitation (REFINE) initiative in Nepal to improve resuscitation competencies using the HBB programme (39). This programme is designed with a combination of theory and simulation sessions with real-time feedback via a high-fidelity neonatal mannequin. With our experience in providing the NRT in Zanzibar, we propose that an effective NRT in resource-limited healthcare settings should be developed by exploring local needs and continuing with refresher training sessions and a train-the-trainer programme.

### Limitation

Our study has several limitations to address. First, our sample of 23 healthcare professionals was small, reducing the generalizability of our findings. Second, we did not collect participants' previous resuscitation experiences and skills that might have influenced the training outcomes. Third, the language barrier might have caused ineffective communication during the courses. Although native speakers facilitated the training, the participants might not have immediately understood relevant statements or instructions by the trainers. This could have led to the loss of potential useful information affecting the evaluation data. Finally, the interval between the first NRT and the refresher training was nine months, which can be considered long. Shorter intervals are recommended. Monthly, quarterly and bi-annual resuscitation refresher courses have been demonstrated to be more effective than longer intervals (40).

## Conclusion

Our study demonstrated overall progress and retention of knowledge and skills in neonatal resuscitation training among a small group of pediatric healthcare professionals from resource-limited settings. Continuous neonatal resuscitation training based on the local conditions and participants' needs has been effective and essential for professionals in Zanzibar. Hence, future research should focus on the short-term and long-term effectiveness of a NRT, including the health outcomes of newborns. Ideally, these studies need to include factors that influence infant mortality, such as prematurity, sepsis, birth asphyxia, and malnutrition. Collaborations between pediatric healthcare professionals and governmental support to improve the competencies of neonatal resuscitation performance will benefit the medical staff, infants, and parents, through both a NRT and training of neonatal health promotion and management. These combined efforts might contribute to reaching the Sustainable Development Goals in reducing neonatal mortality to at least 12 per 1,000 live births.

## Data Availability Statement

The raw data supporting the conclusions of this article will be made available by the authors, without undue reservation.

## Ethics Statement

This study was approved by Medical Ethics Committee of Hunan Children's Hospital (HCHLL-2018-03). Written informed consent for participation was not required for this study in accordance with the national legislation and the institutional requirements.

## Author Contributions

XD, LW, MIM, YH, JQ, SL, MZ, LZ, and JML contributed to the design of the study. XD, MIM, JQ, SL, and MZ contributed to the data collection. XD, YH, and JML contributed to the data analysis. XD and JML drafted the first manuscript. LW, MIM, YH, JQ, SL, and LZ provided revisions. All authors contributed to manuscript revision, read, and approved the submitted version.

## Conflict of Interest

The authors declare that the research was conducted in the absence of any commercial or financial relationships that could be construed as a potential conflict of interest.
