# Supplementary material for: Evaluation of a Neonatal Resuscitation Training Programme for Healthcare Professionals in Zanzibar, Tanzania: A Pre-post Intervention Study
Source: Front Pediatr. 2021 Jun 28;9:693583. doi: 10.3389/fped.2021.693583 (PMC8273261; doi:10.3389/fped.2021.693583)
Supplement: Supplementary file 1 [file Data_Sheet_1.PDF]

Evaluation of a neonatal resuscitation training programme for healthcare professionals in Zanzibar, Tanzania: a pre-post intervention study

Xiang Ding, Li Wang, Mwinyi I. Msellem, Yaojia Hu, Jun Qiu, Shiying Liu, Mi Zhang, Lihui Zhu, Jos M. Latour

Electronic Supplement Material 1: Knowledge Test Questionnaire

Dear Participants：

In order to evaluate the effectiveness of theoretical training, we want to explore your command of knowledge. Please complete the survey before and after the training, tick "√" on the corresponding letter of each multiple choice questions, only 1 answer was correct of the 4 answer options. All the data will be used for research only and conserved as privacy, thanks for your cooperation!

………..

Personal information of participants

1. Name:
2. Gender:
3. Date of Birth:
4. Name of the institution:
5. E-mail:
6. Mobile phone:
7. Profession:
8. Working Experience: less than 3 years, 3 ~ 5 years, 5 ~ 10 years, more than 10 years
9. Education Background:

Theoretical Questionnaire

1. What is the best way to manage the airway inside a hospital when cardiac arrest occurs?

A. Oral-pharynx breather B. Laryngeal mask C. Tracheal intubation D. Tracheotomy

2. Which vital signs must be repeatedly assessed during resuscitation for newborns?

1. Breath<br>2. Blood pressure<br>3. Heart rate<br>4.Complexion

A. 1, 3 B. 2, 3C. 2, 4 D. 1, 4

3. How to check whether positive pressure ventilation is effective?

A. By raising the pressure meter reading to above 30CmH2O

B. And listening to breath sounds.

C. By observing obvious rise and fall of the thorax.

D. By observing improvement of complexion.

4. An auto-inflating air sac is equipped with an oxygen storage device and is connected to an oxygen source with 100% concentration. What concentration level of oxygen can be given to a newborn?

A. 30%~40%   B. 50%~60%C. 70%~80%   D. 90%~100%

5. In the initial few steps of positive pressure ventilation, what should be the respiratory rate (times/minute)?

A. 10~20B. 20~40C. 40~60D. 60~80

6. If an aerocyst face mask is to be used on a newborn for positive pressure ventilation for a long time:

A. Gastric tube must be inserted.

B. The device must be inserted through the mouth, throat and airway.

C. Epinephrine should be given to the newborn.

D. The newborn must be put in the right position.

7. Which of the following does not indicate improvement in a newborn being resuscitated?

A. Higher heart rate   B. Improved complexion

C. Lower muscular tension (muscle relaxing)   D. Autonomous respiration

8. How frequent should positive pressure ventilation and external chest compression be when they are combined together?

A. 40 breaths, 120 compressions   B. 30 breaths, 90 compressions

C. 60 breaths, 120 compressions   D. 20 breaths, 80 compressions

9. How deep should the sternum be compressed during cardiac compression for a newborn?

A. Deep to around 1/3 of the thorax’s anteroposterior diameter

B. Compressed to around 1/3 inch

C. Deep to until you feel the front part of the spine

D. Deep to the area from below the xiphoid to the liver

10. What is the appropriate method of administering epinephrine to newborns in resuscitation?

1. Intravenous
2. Subcutaneous
3. Endotracheal tube
4. Intramuscular

A. 1, 2 B. 2, 4. C.1, 3D. 2, 4

11. What is the dose of expander for a newborn weighing 3000g?

A. 0.3 mL B. 3 mL C. 30 mL D. 300 mL

References:

1.Gomella, TL, Cunningham MD, Eyal FG, Neonatology:Management, Procedures, On-call problems, Disease, and Drugs (six edition).2013, New York: McGraw-Hill Education Medical.

2.Wyckoff MH, Aziz K, Escobedo MB, et al. Part 13: neonatal resuscitation: 2015 American Heart Association guidelines update for cardiopulmonary resuscitation and emergency cardiovascular care[J]. Circulation, 2015, 132(18_suppl_2): S543-S560.
