# Supplementary material for: Evaluation of a Neonatal Resuscitation Training Programme for Healthcare Professionals in Zanzibar, Tanzania: A Pre-post Intervention Study
Source: Front Pediatr. 2021 Jun 28;9:693583. doi: 10.3389/fped.2021.693583 (PMC8273261; doi:10.3389/fped.2021.693583)
Supplement: Supplementary file 2 [file Data_Sheet_2.PDF]

Evaluation of a neonatal resuscitation training programme for healthcare professionals in Zanzibar, Tanzania: a pre-post intervention study

Xiang Ding, Li Wang, Mwinyi I. Msellem, Yaojia Hu, Jun Qiu, Shiying Liu, Mi Zhang, Lihui Zhu, Jos M. Latour

Electronic Supplement Material 2: Skill performance assessment form

NAME ：_________ SCORE：					
Score：0＝undone 1＝incorrect, incomplete, or wrong sequence 2=accurately completed in the correct sequence					
No.	score	Items	0	1	2
1	2	Check resuscitation air bags, masks and oxygen sources			
	2	Ask 4 questions about the newborns:<br>(Term gestational? Amniotic fluid? Breathing or crying? Good tone?)			
2	2	(Optional) If there is Meconium-stained Amniotic Fluid(MSAF), identify the indications for tracheal intubation and suction			
	2	Keep warm			
	2	Position correctly(nasal suction position), clear secretions if needed（suck the mouth before the nasal cavities）			
	2	Wipe dry, remove the wet towel, and re-position			
	2	Stimulate (pat the baby's sole twice)			
	2	Require description of breathing, heart rate and skin tone			
	2	Atmospheric oxygen supply (with spontaneous breathing, heart rate > 100 beats / min)			
3	2	Positive-pressure ventilation indication (Apnea or Gasping breath, heart rate <100 beats / min, with dyspnea or persistent cyanosis after Atmospheric oxygen supply or CPAP)			
	2	Correct positive-pressure ventilation (40-60 times / min)			
	2	Check heart rate improvement (Instruction: no improvement in heart rate)			
	2	The first 5-10 breaths does not show effective ventilation, and the ventilation steps should be corrected(MR.SOPA)<br>(Mask adjustment, raise the jaw, Suction mouth & nose, open the mouth, increase the pressure, Alternative airway)			
	2	Re-evaluate heart rate (guide language: heart rate <60 beats / min)			
4	2	Determine indication for endo-tracheal intubation			
	2	Operate correctly or assist with endo-tracheal intubation			
	2	Correctly confirm the location of the tracheal tube			
5	2	Make sure that chest compressions are necessary (after 30-seconds effective positive pressure ventilation, the heart rate is still <60 beats / min)			
	2	Demonstration of correct chest compression techniques			
	2	The chest compression frequency is correct and matched with ventilation (please exchange the position of the trainees and assistants)			
6	2	Determine the indications for using epinephrine (after 30s positive pressure ventilation combined with chest compressions, heart rate is still <60 times / min)			
	2	Correct dose of epinephrine (0.1 ~ 0.3ml / kg intravenous or 0.5 ~ 1.0ml / kg endo-tracheal tube drip)			
		Prepare for umbilical vein catheterization			
		Insert umbilical vein catheter			
		Inject epinephrine into the umbilical vein catheter or endo-tracheal tube drip			
	2	(optional item) Identify indications for volume expansion			
	2	Correctly describe the name and dosage of commonly used expansion			
End	2	Properly continue/terminate positive pressure respiration or stop oxygen supply			
		Total score: 50 Pass score: 30			
		Total score of trainees			

References:

Ye HM, Yu RJ, Wang DH, et al. Chinese Neonatal Resuscitation guideline. Chin J Appl Clin Pediatr. 2017,32 (14): 1058-1062
